# Prognostic Value of Serum Thyroglobulin and Anti-Thyroglobulin Antibody in Thyroid Carcinoma Patients following Thyroidectomy

**DOI:** 10.3390/diagnostics11112080

**Published:** 2021-11-10

**Authors:** Hashem O. Zahra, Gamal A. Omran, Ahmed G. Gewely, Ahmed Fathy Eldehn, Walied Abdo, Ehab Kotb Elmahallawy, Tarek M. Okda

**Affiliations:** 1 Department of Biochemistry, Faculty of Pharmacy, Damanhour University, Damanhour 22511, Egypt; drhashemzahra90@yahoo.com (H.O.Z.); gamal.omran@pharm.dmu.edu.eg (G.A.O.); Trekokda@pharm.dmu.edu.eg (T.M.O.); 2 Department of Oncology Medicine, Faculty of Medicine, Alexandria University, Alexandria 21111, Egypt; wbodo21734@gmail.com; 3 Department of Otorhinolaryngology, Kasr Al-Ainy Medical School, Cairo University, Cairo 12613, Egypt; ahmedeldehn@kasralainy.edu.eg; 4 Department of Pathology, Faculty of Veterinary Medicine, Kafrelsheikh University, Kafrelsheikh 35516, Egypt; walid.eid@vet.kfs.edu.eg; 5 Department of Zoonoses, Faculty of Veterinary Medicine, Sohag University, Sohag 82524, Egypt

**Keywords:** WDTC, thyroid carcinoma, thyroglobulin, anti-thyroglobulin antibody, thyroid transcription factor 1

## Abstract

Well-differentiated thyroid cancer (WDTC) is a malignant head and neck tumor with a very high incidence. Thyroidectomized WDTC patients have been referred to nuclear medicine for radioactive iodine (RAI) ablation therapy and/or annual follow-up with diagnostic whole-body imaging. Serum thyroglobulin (TG) and thyroglobulin antibodies (TGAb) are biochemical tumor markers used to monitor WDTC. A global rise in the prevalence of WDTC is increasing the number of thyroidectomized patients requiring lifelong monitoring for persistent or recurrent diseases. The present study aimed to identify the most successful prognostic factors in well-defined thyroid carcinoma patients following total thyroidectomy and RAI therapy, followed by an estimation of the cutoff value of TG and TGAb. In this context, a total of 100 subjects were recruited and classified as follows: 60 thyroid carcinoma patients underwent total thyroidectomy and successful RAI therapy, while 40 normal healthy individuals matched for age, sex, and socioeconomic status constituted the control group. Interestingly, the levels of TG did not differ significantly between the relapsed and non-relapsed cases, but the levels of TGAb differed significantly between the relapsed and non-relapsed cases. Collectively, TG and TGAb are considered the most successful prognostic factors in well-defined thyroid carcinoma patients after total thyroidectomy and RAI therapy. The present study also concluded that the TGAb determination was better than that of the TG level, with a cutoff value of 10 ng/mL. These findings provide baseline information for follow-up and lifelong monitoring of thyroidectomized WDTC patients. Further research is warranted to explore more about serum TG and TGAb in thyroid carcinoma patients on a larger scale.

## 1. Introduction

Well-differentiated thyroid cancer (WDTC) is a malignant head and neck tumor with a very high incidence worldwide. The detection rate of thyroid cancer is improving and rising, along with the development of ultrasound technology and the fine-needle aspiration biopsy technique. Papillary, follicular, and mixed carcinomas are considered the most common types of thyroid cancer and have high 5-year survival rates after surgery. However, some patients with thyroid cancer may have distant metastases after surgery, such as bone and pulmonary metastases [1,2]. Despite the obvious development in the checking methods and modern technologies in the diagnosis of these diseases, hepatocellular carcinoma and thyroid cancer are major etiologies of cancer-related deaths [3]. However, it should be borne in mind that it is not easy to evaluate patients who benefit from adjuvant therapy and prevent the development of new agents [4]. For decades, the standard WDTC treatment has involved thyroidectomy and radioactive iodine (RAI) doses, followed by thyroid hormone suppression of thyroid-stimulating hormone (TSH), irrespective of the risk of recurrence [5,6]. According to available literature, thyroidectomized WDTC patients, including those with the papillary, follicular, and papillary–follicular variants, had been referred to nuclear medicine for RAI ablation therapy and/or annual follow-up with diagnostic whole-body imaging. The initial and follow-up evaluation entails the measurement of TSH, serum TG levels, and neck sonogram, before RAI for diagnostic or therapeutic purposes was recommended by the American Thyroid Association (ATA) guidelines [7,8]. The ATA task force guidelines for the evaluation of thyroid cancer have evolved and recommend that patients with WDTC should be classified accordingly into low-, intermediate-, and high-risk groups

It should be stressed that a universal increase in the prevalence of WDTC in thyroidectomized patients requires monitoring of these patients for recurrent or permanent disease through periodic (6–12 months) serum thyroglobulin (TG) estimation and anatomic imaging [9,10]. Patient management is then guided on this stratification according to the recurrence of the disease [11]. It is noteworthy to state that serum TG is considered a well-known tumor-specific marker that is indicative of functional thyroid tissue remnant and/or thyroid cancer residual or recurrent disease. Clearly, TG could be considered a major biochemical marker used to follow WDTC [11]. However, it should be borne in mind that the origin of TG is thyroid tissue proteins and that certain parameters may influence the concentrations of TG [12]. These parameters include the thyroid tissue mass, the efficiency of the thyroid tissue for TG synthesis, secondary injury, the grade of TSH production that affects the level of TG, and the potential of increased thyroglobulin antibody (TGAb) clearance that would modify the concentrations of TG and TGAb. Previous studies on pathological and serological autoimmune thyroiditis reported that TGAb might play an important role in differentiating between thyroiditis and cancer [12,13,14]. However, the role of autoantibodies and the influence of thyroiditis in the pathogenesis of thyroid cancer remain unclear [14]. Patients with WDTC have an approximately 1.5-fold higher rate of occurrence of TGAb than the general population with benign nodules [15]. In addition, TGAb more frequently occurs in papillary thyroid carcinoma (PTC) patients than in patients with follicular carcinomas [16]. The levels of TGAb differ significantly between patients with and without autoimmune thyroid disease (AITD), including those with WDTC. On reviewing the available literature, we found that TGAb-Fab was used for TGAb recognition in patients with non-AITDs (nontoxic multinodular goiter and PTC) and AITDs (hyperthyroidism and goiter disease) [17,18]. It was found that region A was the major immuno-region of TG due to the highest levels of inhibition by the TGAb-Fab of region A in all patients, regardless of AITD [19]. AITD patients had significantly higher inhibition levels than non-AITD patients [13].

Importantly, immunohistochemistry (IHC) is considered an important technique for pathologists in basic research to clarify the pathophysiology of diseases. It should be stressed that thyroid markers, i.e., thyroid transcription factor 1 (TTF1) and TG, enable scientists to characterize approximately 100% of PTCs [20]. However, consideration of the stability of consistency between observers and the objectiveness of interpretation of the results of IHC is crucial. Given the above information, the present study investigates the most successful prognostic factors in well-defined thyroid carcinoma patients following total thyroidectomy and RAI therapy and estimates the cutoff value of TG and TGAb.

## 2. Subjects and Methods

### 2.1. Ethical Approval and Informed Consent Statement

The study was approved by the Research Ethical Committee, Faculty of Pharmacy, Damanhour University, and the ethical approval number is 719PB12. In addition, informed consent was obtained from all participants, and the study was also performed in full accordance with the principle of Good Clinical Practice and according to the guidelines of the Helsinki Declaration.

### 2.2. Sampling

**Figure 1:**
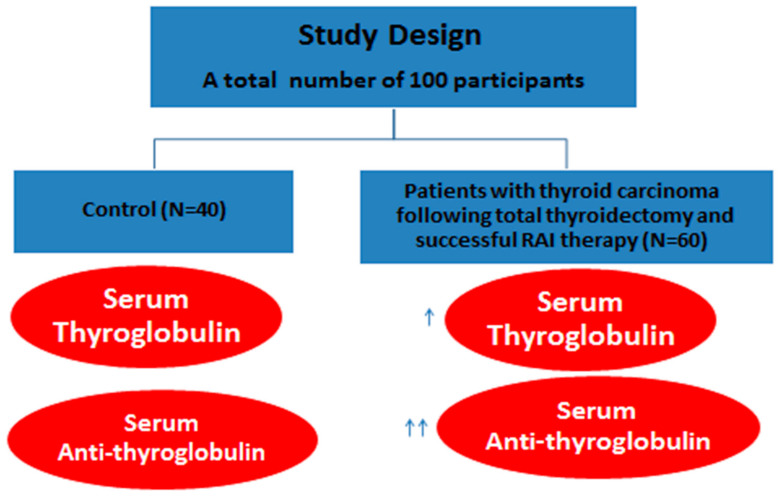
Flow chart of the study procedure. Arrows refer to the increase in the serum level of Tg and anti-TgAb values.

The study included patients who were admitted to the Damanhour Oncology Center in Egypt during the period from October 2019 to May 2020. The experimental design is shown in Figure 1. This study was conducted on 100 individuals: 60 patients with thyroid carcinoma who underwent total thyroidectomy and successful RAI therapy and 40 normal healthy individuals who were matched for age, sex, and socioeconomic status (the control group). Controls were used in the present work to assess the correlation between the thyroidectomized cancer thyroid patients Tg and anti-TgAb values with the normal subjects. Taken into account, participants underwent total thyroidectomy and received levothyroxine replacement therapy. For patients with a negative whole-body scan and therapy with RAI, 5 mL of a fasting blood sample was collected from each participant five days after the last negative RAI: 1-mL aliquots of the whole blood on EDTA were used to perform complete blood counts, while the remaining 4-mL aliquots were centrifuged to separate the sera that were stored at −80 °C until further use.

### 2.3. Inclusion Criteria

WDTC patients (papillary and variants, or follicular) who underwent total thyroidectomy and had successful repeated doses of RAI therapy and negative RAI scans were included. The criteria and cohort for participants in the study, including types and percentage of thyroid carcinoma, are illustrated in Table 1.

### 2.4. Exclusion Criteria

Patients with positive RAI scans indicating residual and/or recurrent disease were excluded. Further evaluation with diagnostic computed tomography (CT), magnetic resonance imaging, or positron emission tomography/computed tomography (PET/CT) was performed for those patients with high TG and/or TGAb and negative RAI scan, and further treatment.

### 2.5. Determination of Serum TG Levels Using the ELISA Technique

The kits used for determining serum TG levels were purchased from HumaCLIA (1304 Langham Greek Dr, Suite 226, Houston, TX, USA). The microtiter plate used in this study was precoated with an antibody specific to TG. A volume of 100 µL standards or samples was added to suitable plate wells (microtiter) with a biotin-conjugated antibody of TG and incubated for 2 h at a temperature of 37 °C. Subsequently, 100 μL of avidin conjugated to horseradish peroxidase was added to each microplate well and incubated for 1 h at 37 °C. Later on, a 100-μL aliquot of the mixture of substrates A and B was added to generate color and incubated for 10 min at 37 °C. Upon plate development, the intensity of the emitted light is proportional to the TG level in the sample or standard at 450 nm; the TG concentration was calculated from the standard curve.

### 2.6. Determination of Serum TGAb Levels Using the ELISA Technique

The ELISA kits were purchased from Diagnostic Automation, Inc. (23961 Craftsman Road, Suite D/E/F, Calabasas, CA, USA). Purified TG antigens were coated on the surface of the microwells. Samples, standards, and blanks were diluted in a ratio of 1:40, and a 100-µL aliquot was added to the wells and incubated for 30 min; the TG-specific antibodies were bound to the antigens. All unbound materials were washed three times. After adding 100 µL of enzyme conjugate, anti-human IgG was bound to the antibody–antigen complex and incubated for 30 min. Excess enzyme conjugates were then washed three times, and TMB chromogenic substrate was added. A total volume of 100 µL of the enzyme conjugate was added, and the catalytic reaction was stopped. The intensity of the color produced is directly proportional to the IgG-specific antibodies present. The absorbance was then read by a microwell reader at 450 nm and compared with standards and blanks.

### 2.7. Immunohistochemistry

In this step, TG and TTF1 were determined by immunohistochemistry for the paraffin blocks from tissues, which were prepared after proper handling of the specimens and fixation as described elsewhere [21]. Immunohistochemical studies were then performed on 4-µm thick, formalin-fixed, paraffin-embedded tissue sections through the following methods. 

#### 2.7.1. Immunohistochemical Detection of TTFI

Anti-TTF1 antibody (EPR8190-6) ab133638 was purchased from Abcam (Discovery Drive, Cambridge Biomedical Campus, Cambridge, UK). In addition, the following kits were purchased: rabbit monoclonal (EPR8190-6) to TTFI (low endotoxin, Azide-free). The slides were then washed twice for 5 min in TBS plus 0.025% Triton X-100 with gentle agitation, then blocked in 10% normal serum with 1% BSA in TBS for 2 h at room temperature and drained for a few seconds, and wiped around the sections with tissue paper. Then, heat-mediated antigen retrieval with Tris/EDTA buffer (pH 9.0) was performed before commencing with the IHC staining protocol. Primary antibodies were diluted in TBS with 1% BSA and incubated overnight at 4 °C. The slides were rinsed twice for 5 min in TBS 0.025% Triton with gentle agitation and incubated in 0.3% H_2_O_2_ in TBS for 15 min. Enzyme-conjugated secondary antibody was applied to the slide, diluted in TBS with 1% BSA, and incubated for 1 h at room temperature. Then, the slides were developed with chromogen for 10 min at room temperature and rinsed with running tap water for 5 min. The slides were then dehydrated, cleared, and mounted.

#### 2.7.2. Detection of TG Immunohistochemically in the Tissue after Total Thyroidectomy

Anti-TG antibody (EPR9730) (low endotoxin, Azide-free, ab229449) was purchased from Abcam (Discovery Drive, Cambridge Biomedical Campus, Cambridge, UK). The following kits were purchased: rabbit monoclonal (EPR9730) to TG (low endotoxin, Azide-free). Whole-body scans with RAI were performed after total thyroidectomy and successful RAI treatment to exclude positive patients.

### 2.8. Statistical Methods

The statistical analysis was carried out using IBM SPSS statistics program version 21 and R software. Quantitative data were described by mean and median as measures of central tendency, as well as standard deviation, minimum, and maximum as measures of dispersion. Chi-square test was used to study significant association between two categorical variables. Fischer exact, as well as Monte Carlo significance was used if more than 20% of total expected cell counts were <5 at 0.05 level of significance. Independent sample *t*-test was performed to compare mean TG and TGAb between cases and controls. Mann–Whitney test was performed to detect significant difference in the median quantitative variables (TG and TGAb) between two groups of patients with and without relapse. The choice of tests depended on distribution of variables by Kolmogorov Smirnov test. Receiver operation coefficient (ROC) curve analysis was carried out to detect the predictive accuracy of thyroglobulin and anti-thyroglobulin for relapse among cases. Area under the curve (AUC), sensitivity, specificity, positive predictive value, and negative predictive values were used to evaluate each index, where sensitivity (SE) = true positive/true positive + false negative, specificity (SP) = true negative/true negative + false positive, positive predictive value (PPV) = true positive/true positive + false positive, and negative predictive value (NPV) = true negative/true negative + false negative. AUC was interpreted as 0.90–1 = excellent, 0.80–0.90 = good, 0.70–0.80 = fair, 0.60–0.70 = poor, 0.50–0.60 = fail. All statistical tests were judged at 0.05 significance level.

## 3. Results

**Figure 2:**
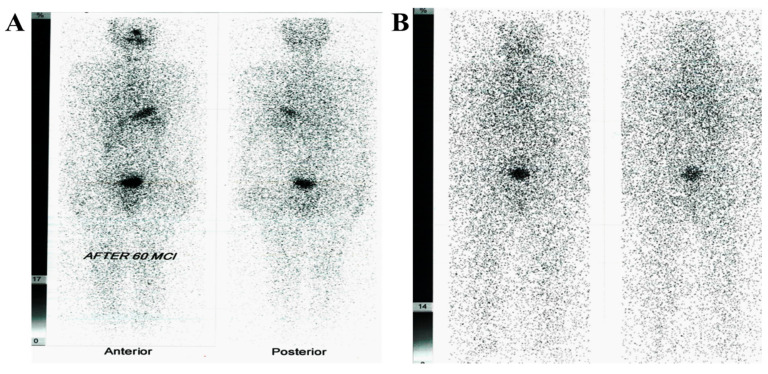
(**A**) ^131^I whole-body scan. The study was performed 5 days post oral administration of 60 mCi, ^131^I therapy dose showing Manuel tracer uptake in the neck at the thyroid bed region with no abnormal tracer accumulation in the rest of the body. (**B**) ^131^I whole-body scan. The study was performed 48 h post oral administration of 5 mCi, ^131^I showing no active thyroid tissues, either in the neck or in the rest of the body (negative study).

All patients underwent total thyroidectomy, followed by successful treatment with RAI therapy (^131^I), and then negative whole-body scans with 3 mCi ^131^I (Figure 2). Importantly, 7 of the 60 patients experienced relapse and had positive PET/CT. RAI scans were then performed 5 days after treatment for all patients. Table 2 shows the levels of Tg and anti-TG among study participants (cases and control). The level of TSH values in control and thyroidectomized patients are depicted in Table 3. Meanwhile, Table 4 presents a significant increase in the level of serum transaminase (ALT and AST) in the thyroid carcinoma group compared with the control group. As regards CBC indices, the results showed a significant decrease in the hemoglobin concentration, platelet count, and white blood cell count in the carcinoma group.

**Figure 3:**
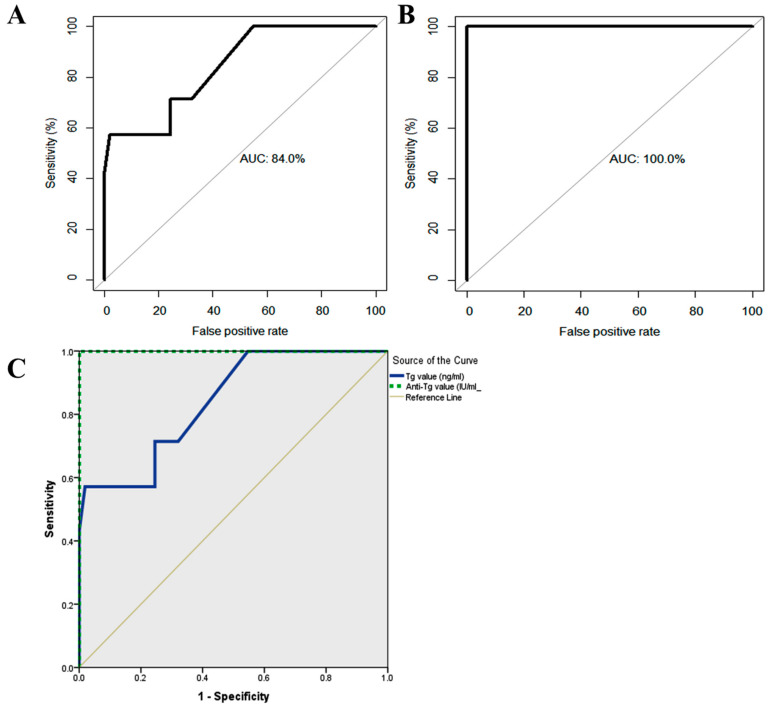
(**A)** Predictive performance of TG values for relapse outcome in patients with thyroid cancer. (**B**) Predictive performance of anti-TG for relapse outcomes in patients with thyroid cancer. (**C**) Comparison of the predictive performance of TG and anti-TG for relapse outcomes.

**Figure 4:**
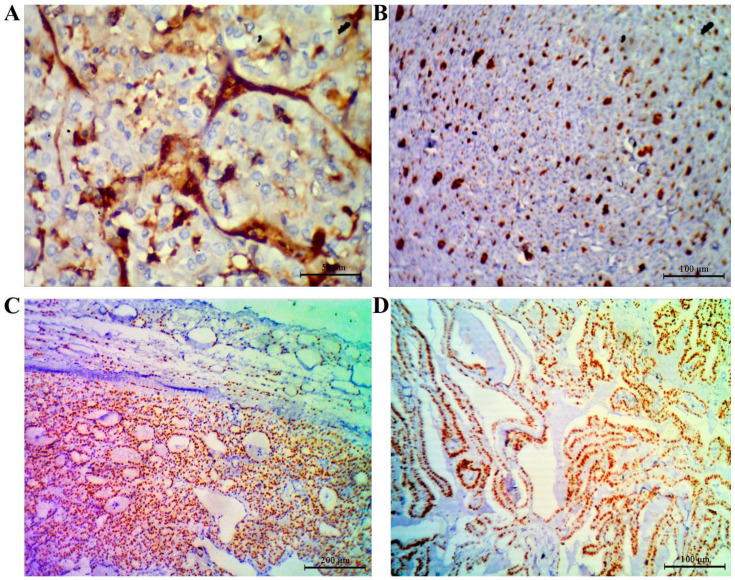
(**A**) Positive thyroglobulin in papillary carcinoma. (**B**) Positive thyroglobulin in follicular carcinoma. (**C**) Positive TTF1 in follicular carcinoma showing vascular invasions. (**D**) Positive TTF1 in papillary carcinoma.

On the other hand, the results showed nonsignificant differences in the neutrophil and lymphocyte counts between the studied groups. Table 5 shows the predictive performance of tissue TG, as well as anti-TG for the prediction of relapse among thyroid carcinoma cases. A cutoff value of ≥0.350 for TG best predicted a relapse, with a corresponding accuracy of 93.3 (95% confidence interval (CI), 93.1–93.5%). Clearly, TG could be a very good predictor of relapse, as denoted by 0.84 AUC (95% CI, 0.679–1.0%). The specificity of TG is 98.1% (95% CI, 94.5–100%), which is greater than the sensitivity (57.1%; 95% CI, 20.5–93.8%). This means that TG is a good positive test rather than a good negative test, with a corresponding higher NPP than PPV (Figure 3A). However, the predictive performance reveals that anti-TG is a perfect predictor of relapse, with an AUC of 1 (95% CI, 1–1%), accuracy of 100%, sensitivity of 100%, specificity of 100%, PPV of 100%, and NPV of 100% (Figure 3B). The comparison of areas under the ROC curves revealed that anti-TG had a significantly better predictive performance than TG (*p* = 0.048) (Figure 3C). Figure 4A–C shows that all patients were positive for TTF1 and positive for tissue TG (papillary and follicular) by histopathological examination. 

In according with IHC, Table 6 depicts the distribution of the patients according to the recorded immunohistochemical findings and the pathology regarding TG and TGAb. As shown, 23 out of 60 WDCT tested patients experienced initial abnormal pathological findings, including capsular invasion, vascular invasion, and LN metastasis–central LNs. Meanwhile, the remaining patients (N = 37) had WDCT with, noncapsular, nonvascular invasion and non-LN metastases. In addition, there was no significant difference (*p* > 0.05) between the reported pathological findings (capsular invasion, vascular invasion, and LN metastasis) and their corresponding Tg and TgAb results.

## 4. Discussion

The last few decades have witnessed a global rise in the prevalence of WDTC in thyroidectomized patients, which highlights the necessity of lifelong monitoring for persistent or recurrent diseases [22,23]. The present study provides interesting findings on the potential prognostic value of serum TG and anti-TGAb in thyroid carcinoma patients after thyroidectomy. The study also revealed the role of IHC in the detection of abnormal pathological findings in WDCT patients.

As shown in our results, according to ^131^I ablative therapy or diagnostic ^131^I scanning, a significant proportion of patients (20.2% and 8.3%, respectively) had residual benign or malignant thyroid tissue/metastatic diseases on whole-body scanning, despite a negative stimulated serum TG level. Our results are similar to those of previous studies, suggesting that several of the currently available immune radiometric assays are not sensitive to detect low levels of serum TG [24]. Several possible factors might reduce the sensitivity of the TG assay, such as the heterogeneity in the diffusion of TG, involvement of the heterophile antibody, and the “hook” effect. Conventional competitive radio-immunoassays were used to determine serum TG levels, which have been replaced by noncompetitive immunoassays based on antibody tracer-labeled I-125. It is noteworthy to state that TG is present in different isoforms, with an increase in heterogeneity between patients with thyroid cancer due to the uncontrolled biosynthesis [25]. Immunoassays relied on various monoclonal antibodies with different TG epitopes, and the heterogeneity of isoforms contributes to the variability and false-negative results of serum TG [26]. Endogenous TGAb is also a known marker of serum TG analysis, with a relationship between a high level of serum TGAb and a low level of serum TG [27]. The determination of TGAb levels is based on immunoassays that are attached to the endogenous TGAb epitopes, which vary between individuals. Not all TGAbs were detected, resulting in a false-negative result [28]. Taking all these findings into account, the threshold level of the assay is considered negative or positive for TGAb, depending on the type of technique used. The normal TGAb detection limit is primarily cited for certain immunoassays. However, it was reported that, even when TGAb has a normal value, it might adversely impact the TG level, and patients should only be considered TGAb-negative when the antibody is completely absent [29]. When a stricter threshold for the undetectable TGAb signal was used in our post-ablative ^131^I group, there was still a significant proportion of patients (9.3%) who fell into the category of being TG/TGAb-negative or ^131^I scan-positive, suggesting a truly false-negative TGAb or TG result. These findings reflect that the presence of serum TGAb, even when below the upper limit of normal, may still be associated with the interference of TG radio-immunoassays. 

Importantly, several studies have shown an obvious link between the mass of thyroid tissue and the level of TG, which means lower volumes of thyroid tissue are associated with lower stimulated TG levels [30]. Whole-body ^131^I scanning may be very sensitive in detecting the too-small thyroid tissue that produces minute amounts of TG, particularly post-ablation [31]. The present findings reveal a correlation between TGAb levels and recurrence, which is in harmony with a previous study compared the antithyroglobulin antibody concentrations before and after ablation with 131I as a possible predictor of structural disease in differentiated thyroid carcinoma patients TgAb [32]. In a previous study, patients exhibited very low levels of Tg and positive TgAb after (131)I ablation, and the frequency of disease in patients was <5%, with >50% reduction in TgAb [32], suggesting the major advantages of imaging methods other than ultrasound (US) during initial assessment and long-term follow-up [32]. In contrast to our present findings, some previous studies [33,34] reported no correlation between TGAb levels and recurrence, which might be attributed to the fact that Tg and anti-Tg vary from one center to another, with minor differences in relation to the sensitivity and accuracy. The other possible explanation for discrepancy in results might include the size of cohort and that the recurrence was only observed in seven patients, besides the confounding factors of residual thyroid. Clearly, the designing of large cohort studies would be very helpful for better understanding of the prognosis. 

Taken into consideration, the hook effect is a common feature in most immunoassays and occurs in the presence of high antigen levels. It results in a binding capacity of antibodies for solid support; therefore, it causes a false-negative result [35]. The hook effect may affect the TG and TGAb levels; however, in the case of TG measurements in the present study, this is considered very unlikely as post-ablative and diagnostic ^131^I patients would not be expected to have high TG levels in the post-thyroidectomy or ablative ^131^I. The hook effect may have potentially played a role in a false-negative TGAb result. It was demonstrated that neoplastic cells may still be present in the lymph nodes that are visible even after several treatments with ^131^I whole-body scans, but in an amount that is too small to produce a measurable serum TG level [36,37]. In our study, the TG levels were not significantly increased in relapsed cases, which mean no significant differences were observed between the relapsed and non-relapsed patients, which may be due to the interference of TGAb. Despite all relapsed patients having a negative RAI scan and undetectable TG levels, their TGAb levels were high and their PET/CT was positive. In addition, some of the patients had nonsignificant detectable TG levels. Clearly, our study found that TGAb is more specific for cancer relapse for WDTC than TG, which might be attributed to the antibody interference or the hook effect. Moreover, the cutoff or threshold values for TG in patients with WDTC after total thyroidectomy had no significant value in our present work, and no significant differences were reported between the relapsed and non-relapsed patients. Additionally, our results revealed that the best test for relapse is PET/CT. However, it should be borne in mind that PET/CT is an expensive and time-consuming test, but it is considered the best practical prognostic test for TGAb. 

Importantly, IHC is considered a good tool for discovering the efficacy of new biomarkers used in personalized medicine [38,39]. Taken into consideration, IHC procedures that have been automated since the improvement of this technique are particularly important for newly discovered molecules and/or antibodies. As shown, our study depicted that tissue TG and TTF1 were positive in all patients with WDTC. These results confirm that tissue TG and TTF1 could be accurate diagnostic factors in thyroid carcinoma patients after thyroidectomy. On an important note, the specificity and sensitivity of IHC should be checked [40] and the interpretation of IHC needs to be carefully planned [41].

## 5. Conclusions

Given the above information, the present study concluded on the prognostic value of serum TG and TGAb in thyroid carcinoma patients after thyroidectomy. The present study also revealed that serum TGAb level determination was better than that of serum TG level as a prognostic factor to check for relapse in WDTC patients after total thyroidectomy. The limitations of the present work include the small sample volume, since the cohort only included a small number of patients with recurrence. Further future research is warranted to explore more about serum TG and anti-TGAb in thyroid carcinoma patients with recurrence on a larger scale.

## Figures and Tables

**Table 1:** Full details of the study cohort for participants, including types and percentage of thyroid carcinoma.

Type of Thyroid Carcinoma	Number of Cases	Percentage (%)
Papillary carcinoma and its variants	52	86.6%
Follicular carcinoma	7	11.6%
Hurthle cell type	1	1.6%

**Table 2:** Comparison of the levels of Tg and anti-TG among cases and controls.

Parameters	Control (*n* = 40)	Cases (*n* = 60)	Significance
	Mean (SD)		
Thyroglobulin (ng/mL)	15.07 (2.13)	23.65 (0.36)	*p* < 0.001
Anti-TG (IU/mL)	58.96 (2.45)	136.84 (6.33)	NS

t: independent sample *t*-test, *p* ≤ 0.05 are significant.

**Table 3:** Comparison between TSH values in normal healthy control and thyroidectomized patients.

	Control	Patients
TSH (Mean ± SD) (µIU/mL)	2.3 ± 1.1	0.96 ± 0.15

**Table 4:** Mean ± SD of liver enzymes and CBC indices in the different studied groups.

Item	Mean ± SD		*p* Value
	Control	Thyroid Carcinoma	
ALT (u/L)	23.76 ± 5.63	33.50 ± 4.93 *	*p* < 0.001
AST (u/L)	25.85 ± 5.39	36.85 ± 5.11 *	*p* < 0.001
Hb (g/dL)	12.27 ± 0.60	11.04 ±.74 *	*p* < 0.001
Hct	41.30 ± 60.61	38.41 ± 2.59	*p* = 0.187
RBCs (10^6^)	5.30 ± 6.06	4.31 ± 0.46	*p* = 0.213
Platelets (10^3^)	259.86 ± 57.42	214.93 ± 41.83 *	*p* < 0.001
WBCs (10^3^)	6.26 ± 1.41	5.34 ± 1.02 *	*p* < 0.001
Neutrophils	54.00 ± 5.46	51.96 ± 5.23	*p* = 0.065
Lymphocytes	34.00 ± 5.46	36.03 ± 5.23	*p* = 0.076

ALT: alanine aminotransferase, AST: aspartate aminotransferase, Hb: hemoglobin, Hct: hematocrit, RBCs: red blood cells, WBCs: white blood cells. * Significance from control group *p* < 0.001.

**Table 5:** The predictive performance of thyroglobulin and anti-TG for diagnosis of relapse among cases of thyroid cancer.

	Cutoff Point	AUC (SE) *p* Value	AUC (95% CI)	Accuracy (95% CI)	SN (95% CI)	SP (95% CI)	PPV (95% CI)	NPP (95% CI)
TG	≥0.350	0.84 ± 0.08 *p* = 0.002 *	(0.679–1.0)	93.3% (93.1–93.5%)	57.1 (20.5–93.8)	98.1 (94.5–100)	80 (44.9–100)	94.5 (88.5–100)
TGAb	≥105.5	1(0) *p* < 0.001 *	(1–1)	100% (100–100%)	100 (100–100)	100 (100–100)	100 (100–100)	100 (100–100)
Comparison of area under the ROC curve between both parameters AUC1–AUC2				difference: areaA − areaB − 0.16 SE of the difference = 0.0962 Z = −1.6625 One-tailed *p* value 0.048 *				

areaA: area under the ROC curve for TG, areaB: area under the ROC curve for anti-TG. SE: standard error, AUC: area under the ROC curve, SN: sensitivity, SP: specificity, PPV: positive predictive value, NPP: negative predictive value. * Significance *p* ≤ 0.05.

**Table 6:** Distribution of the patients according to the recorded pathology regarding Tg and anti-Tg.

Pathology	Thyroglobulin (ng/mL)				Anti-TG (IU/mL)						
	Tg < 1		Tg 1–2		Anti-Tg 5–10		Anti-Tg 10–20		Anti-Tg > 20		
	No.	%	No.	%	No.	%	No.	%	No.	%	*p* Value
Capsular invasion (N = 7/60)	5	27.8	2	40	6	33.3	1	33.3	0	0	
Vascular invasion (N = 10/60)	9	50	1	20	8	44.4	1	33.3	1	50	0.98
LN metastasis (N = 6/60) Total 23/60	4 18	22.2 100	2 5	40 100	4 18	22.2 100	1 3	33.3 100	1 2	50 100	

## Data Availability

The data that support the findings of this study is contained within the article.
